# Microcirculatory changes during goal-directed or mean arterial pressure-guided fluid therapy in abdominal surgery

**DOI:** 10.1186/cc12146

**Published:** 2013-03-19

**Authors:** SP De Wolf, J Stens, RJ Van der Zwan, N Koning, C Boer

**Affiliations:** 1 VU University Medical Centre, Amsterdam, the Netherlands

## Introduction

This study compared the effect of pulse pressure variation (PPV) and cardiac index (CI)-guided fluid therapy versus mean arterial pressure (MAP)-guided fluid therapy on microcirculatory perfusion in patients undergoing abdominal surgery.

## Methods

Patients undergoing elective abdominal surgery were randomized into a PPV/CI-guided group (*n = *11) or a MAP-guided (*n = *12) group. PPV, CI and MAP were measured using the non-invasive finger arterial blood pressure measurement device ccNexfin (Edwards Lifesciences BMEYE, Amsterdam, the Netherlands). Tidal volumes were ≥8 ml/kg with PEEP ≥8 mmHg. In both groups, MAP of 70 mmHg was maintained. In the PPV/CI group, an intraoperative algorithm was used keeping the PPV under 12% and CI above 2.5 l/minute/ m^2 ^using fluid therapy and dobutamine and noradrenaline infusion, respectively. Sublingual microvascular perfusion was measured after anesthesia induction, and every subsequent hour using sidestream dark-field imaging (Microscan; Microvision Medical, Amsterdam, the Netherlands). The perfused small vessel density (PVD) values were offline quantified.

## Results

The first hour during surgery, the PPV/CI-guided group tended to receive more fluids than the MAP-guided group (1,014 ± 501 ml vs. 629 ± 463 ml; *P *= 0.07). At this time point, the PVD was slightly lower in the PPV/CI-guided group (16.7 ± 3.1 mm/mm^2^) when compared with the MAP-guided group (17.9 ± 3.9 mm/mm^2^; *P *= 0.41). In both groups the PVD remained stable during the first 2 hours of surgery. However, 2 hours after the start of surgery, the PVD in the PPV/CI group restored and tended to be higher than in the MAP-guided group (21.1 ± 1.9 vs. 18.1 ± 3.4 mm/mm^2^; *P *= 0.09). After 1 hour of surgery, the administered fluid volume correlated inversely with PVD (*r *= -0.59, *P *= 0.011).

## Conclusion

Goal-directed fluid management resulted in a higher administered fluid volume in the beginning of surgery, and this was associated with a slightly reduced microcirculatory perfusion when compared with MAP-guided fluid management. Microcirculatory perfusion tended to improve as surgery progressed in the goal-directed fluid therapy group. Our findings suggest that goal-directed and MAP-guided fluid management are associated with distinct patterns in fluid resuscitation, which may be of consequence for microvascular perfusion.

